# *Clostridioides difficile* Stimulates *CCL20* Expression in Human Colonoid Monolayers in a Transwell‐Based Coculture System That Supports Its Anaerobic Growth

**DOI:** 10.1155/ijm/1132113

**Published:** 2026-07-07

**Authors:** Paola Zucchi, Adrianne D. Gladden, Andrew W. Day, Jules Dressler, Revathi Govind, Mohammad Almeqdadi, Jatin Roper, Albert Tai, Rebecca Batorsky, Carol A. Kumamoto

**Affiliations:** ^1^ Department of Molecular Biology and Microbiology, Tufts University, Boston, Massachusetts, USA, tufts.edu; ^2^ Graduate School of Biomedical Sciences, Tufts University, Boston, Massachusetts, USA, tufts.edu; ^3^ Division of Biology, Kansas State University, Manhattan, Kansas, USA, k-state.edu; ^4^ Department of Transplant Surgery, Tufts Medical Center and Tufts University School of Medicine, Boston, Massachusetts, USA, tufts.edu; ^5^ Department of Medicine, Duke University School of Medicine, Durham, North Carolina, USA, duke.edu; ^6^ Department of Immunology and TUCF Genomics Core, Tufts University, Boston, Massachusetts, USA, tufts.edu; ^7^ Data Intensive Studies Center, Tufts University, Medford, Massachusetts, USA, tufts.edu

**Keywords:** CCL20, *Clostridioides difficile*, coculture, colonoids, toxins

## Abstract

The pathogenic bacterium *Clostridioides difficile* is a major cause of antibiotic‐associated diarrheal disease. Treatment of the disease is challenging because antibiotics used for treatment may also perpetuate the conditions that contributed to initial susceptibility. Elucidating the mechanisms of *C. difficile*/intestinal epithelium interaction is needed to facilitate the development of new therapeutic options. The studies described in this communication demonstrate the development of a tissue culture system that supported the growth of *C. difficile* in coculture with a model of the human intestinal epithelium produced from colonoids, organoids derived from human colonic biopsies. Epithelial cell responses to *C. difficile* included upregulation of *CCL20*, encoding a chemokine that was previously shown to be upregulated in response to *C. difficile* challenge. Additionally, bacteria associated with the monolayer in a nontoxin‐dependent manner. This system will support future investigation of epithelium/*C. difficile* interactions during CDI and identification of mechanisms that drive pathogenesis by *C. difficile* in the human intestine.

## 1. Introduction

*Clostridioides difficile* infection (CDI) is a leading cause of antibiotic‐associated diarrheal disease and a major source of morbidity and mortality worldwide, particularly among hospitalized and elderly patients [1]. CDI most commonly occurs in individuals recently treated with antibiotics in part because antibiotics disrupt the gut microbiome, reducing the diversity and abundance of resident bacterial taxa that provide colonization resistance against *C. difficile* spore germination and vegetative growth.

Various approaches have been used therapeutically to treat this debilitating infection, yet none are completely successful. The two primary antibiotics used for CDI treatment, vancomycin and fidaxomicin [2], are not 100% effective, and many patients suffer from recurrence of infection after completion of treatment. Newer approaches include fecal microbiota transplantation (FMT), in which an entire microbial community is transplanted into a patient, as an approach for preventing recurrent CDI [3]. However, rare adverse effects have been noted with FMT [4], prompting efforts to develop more targeted microbial interventions. In murine models, introduction of a single bacterium, *Clostridium scindens*, or a small consortium of bacteria reduced *C. difficile* disease [5, 6]. Feeding mice a diet high in plant polysaccharides reduced the ability of *C. difficile* to persist within the GI tract [7], suggesting that dietary approaches could contribute to lowering CDI incidence. Together, these observations highlight the continuing need for new and improved therapeutic approaches to combat CDI.

*Clostridioides difficile* is a strict anaerobe that persists in aerobic environments as a dormant spore. Following ingestion, these spores encounter germinants, such as the bile acid taurocholate, within the gastrointestinal (GI) tract [8]. Germination triggers the emergence of vegetative cells, which proliferate in the anaerobic parts of the GI tract and secrete glucosylating toxins, TcdA and TcdB [9], the primary virulence factors of *C. difficile*. Interactions between the organism, its toxins, and the intestinal epithelium ultimately shape the progression and severity of disease.

One approach for characterizing *C. difficile*/epithelial interactions is to coculture bacteria and epithelial cells in tissue culture. A variety of in vitro systems have been used previously to study the effects of *C. difficile* bacteria or its toxins [10–16]. Some studies have relied on specialized apparatus to generate anaerobic conditions to support *C. difficile* survival and growth; others have used bacterial components or short‐term culture with bacteria [17–20]. The goal of the present study was to develop a simple and accessible coculture system that does not require specialized equipment yet supports robust growth of *C. difficile* alongside epithelial cells. To achieve this, we utilized organoids established from adult colonic biopsies, termed colonoids [21, 22]. Colonoids are derived from human stem cells and thus can be propagated in culture but are not transformed [23]. Colonoids can be differentiated in vitro and generate model epithelia that recapitulate many features of the human colonic epithelium.

Organoids in culture generate hypoxic environments [23]. We, therefore, tested the ability of an epidemic strain of *C. difficile* to grow in coculture with a differentiated colonoid monolayer produced on a transwell and incubated in an aerobic atmosphere. Transwells were used so that the apical surface of the differentiated monolayer was accessible for bacterial interaction. Results showed that bacteria proliferated in this system and maintained viability for at least 20 h. We detected an association of bacteria with the monolayer that increased over time. The epithelial cells responded to bacterial toxin production by upregulating the chemokine CCL20, reproducing a response that has been previously demonstrated [24]. This system provides a simple approach that will enable further investigation of *C. difficile*/epithelial cell interaction.

## 2. Materials and Methods

### 2.1. Bacterial Growth and Spore Preparation

*Clostridioides difficile* strain UK1 (a NAP1/027/BI human epidemic strain [25]) was used for all experiments except as noted.For studies of nontoxigenic *C. difficile*, R20291 (a NAP1/027/BI human epidemic strain closely related to UK1 [26]) and an R20291::*tcdR* mutant strain [27] were used. Lack of TcdA/TcdB production by the *tcdR* mutant strain was verified by testing culture supernatants with the tgcBIOMICS Clostridium difficile Toxin A or B ELISA kit (TGC‐E002‐1).

Bacteria were cultured on prereduced BHIS medium at 37°C in an anaerobic chamber. For sporulation, cultures were grown in prereduced TY broth and plated on prereduced 7030 medium [28]. After 1 week, bacterial growth was collected from the plates. For some experiments, the suspension was incubated in water at 4°C overnight. Spores were collected by centrifugation at 15,000 rpm for 15 min in a Hitachi R20A2 rotor. Spores were washed with water and purified by centrifugation in a discontinuous Histodenz (Sigma D2158) gradient as described previously [29]. Purified spores were washed with water, aliquoted, and stored at −20°C.

For infections, spores were pregerminated by treatment with germinants under aerobic conditions, followed by addition to the colonoid monolayer system, where vegetative cells would outgrow when anaerobic conditions were encountered. A spore aliquot was thawed, diluted to approximately 5 × 10^7^ spores/mL, and incubated at 60°C for 10 min. Spores were diluted with an equal volume of 120 mM glycine (Sigma G7126) and 4 mM sodium cholate (Sigma C9282) in PBS and incubated at 37°C for 15–30 min to produce pregerminated spores. The pregerminated spores were either added directly to epithelial cell monolayer cultures or were collected by centrifugation (Eppendorf 5415 centrifuge, 13,200 rpm, 5 min) and resuspended in the same volume of PBS before addition to monolayer cultures. Ten microliters of pregerminated spores (approximately 5 × 10^5^ spores) were added per 6.5‐mm‐diameter transwell (Costar 3413).

### 2.2. Preparation of Colonoid Monolayers on Transwells

Colonoid line CJ50 was derived from colonic biopsies obtained from a 67‐year‐old Chinese female undergoing routine outpatient colonoscopy for colorectal cancer screening at Tufts Medical Center (IRB #11652, principal investigator: Jatin Roper, date of approval: 8/18/2025). The cells were propagated in Matrigel (Corning 356231) as described previously [30]. Medium composition was as described [22] except that L‐WRN‐conditioned medium (L‐WRN cells ATCC CRL‐3276 [31]) was used at 65% instead of L‐WNT3A‐, R‐spondin‐, and noggin‐conditioned media. Primocin (InvivoGen ant‐pm‐1) was used instead of pen‐strep, and 5 *μ*M Y27632 (Rho kinase inhibitor, Sigma Y0503) was added for the first 48 h of culturing.

For preparation of monolayers on transwells, colonoid spheres were recovered from Matrigel, washed with PBS, and digested with TrypLE Express (Gibco 12605‐010) for 2.5 min at 37°C. After digestion, cells were diluted with 1 volume advanced DMEM (Invitrogen 12634‐028), 1% GlutaMAX (Gibco 35050‐061), and 20% FBS (Fisher 26140079) and filtered through a 40‐*μ*m cell strainer. Cells were diluted with 0.4% trypan blue, counted with a Corning CytoSMART counter, and diluted to 2.5 × 10^6^ cells/mL with warmed 65% L‐WRN medium containing Y27632.

Matrigel was diluted with CMGF‐ (advanced DMEM medium containing additives but not growth factors [22]) to 33 or 66 *μ*g protein/mL, and 125 *μ*L was applied to each 6.5‐mm‐diameter, 0.4‐*μ*m pore, polycarbonate filter transwell (Costar 3413) and incubated at 37°C for 2 h. Liquid was removed from the transwell, the basolateral compartment was filled with 0.5 mL 65% medium, and 50 *μ*L of medium was added to the apical compartment. Cells (2.5 × 10^5^ cells per 6.5‐mm‐diameter transwell) were added, and the seeded transwells were incubated in a tissue culture incubator with 5% CO_2_ at 37°C.

The next day, transwells were given fresh 65% L‐WRN medium containing 5 *μ*M Y27632 (Sigma Y0503), replacing the basolateral medium and adding 0.2 mL to the apical compartment. The next day, the medium was changed to 65% without Y27632 (0.4 mL apically and 0.5 mL basolaterally) and was changed again after 2 days when the monolayer was typically confluent.

Confluent monolayers were differentiated by washing with CMGF‐, followed by feeding with differentiation medium (DM) [22, 32] containing Primocin. Medium was changed the next day with DM containing Primocin. Monolayers were fed 2 days later (Day 3) (DM without Primocin) and in the late afternoon of Day 4 or in the morning of Day 5 (DM without Primocin). Monolayers were inoculated with pregerminated spores on Day 5.

For measurement of *trans*‐epithelial electrical resistance (TEER), a Millicell ERS‐2 volt/ohm meter was used following the manufacturer′s protocol.

### 2.3. Coculturing *C. difficile* With Colonoid Monolayers

Pregerminated spores (approximately 5 × 10^7^/mL) were prepared as above, and 10 *μ*L of the preparation was added gently to the apical medium of the transwell. Control samples were inoculated with 10 *μ*L of buffer. The plates were wrapped with Parafilm and incubated in a standard tissue culture incubator (37°C, 5% CO_2_, room air).

At various times, transwells were taken into the anaerobic chamber (two transwells at a time) to estimate the number of bacteria per transwell. The first 170 *μ*L of apical medium was carefully removed. This medium was not turbid and was discarded. The second approximately 170 *μ*L of medium was collected into the pipette tip and used to wash the monolayer twice and then collected into a preweighed tube. For inoculated transwells, this sample was typically turbid. Ten microliters of this sample was immediately diluted with 1 mL of prereduced PBS to measure CFU. The remainder of the sample was removed from the chamber and weighed to determine the volume recovered from each transwell after correction for the 10 *μ*L removed for CFU.

Transwell monolayers were removed from the chamber and fixed with 4% paraformaldehyde for 15 min at room temp, followed by washing three times with PBS at room temperature. Transwells were stored at 4°C in PBS.

For some experiments, transwells were inoculated with vegetative *C. difficile* cells. Strains were grown in prereduced TY broth and harvested during log phase. A sample of each culture was diluted 1/50 into prereduced PBS. DM medium was prereduced, and 0.4 mL was transferred to a gasketed screw cap tube for each inoculation. Ten microliters of bacteria diluted in PBS was added to each tube (or PBS only for mock inoculation). The caps were screwed on tightly, and the tubes were removed from the chamber. The transwells were inoculated by removing their apical medium and replacing it with the bacteria‐containing medium. Transwells were inoculated with approximately 5 × 10^4^ CFU.

### 2.4. Detection of Secreted Protein Production by Colonoid Cells

In some cases, after incubation of monolayers with *C. difficile* for 16 h, the cultures were treated with Brefeldin A (Sigma B6542) (25 *μ*g/mL final added to the apical and basolateral medium) for 4 h. This treatment was used to block protein secretion and facilitate the detection of the secreted protein CCL20.

### 2.5. Preparation of Anti–*C. difficile* Antiserum

UK1 bacterial cells were grown to the exponential phase in TY broth. Cells were washed three times with PBS, resuspended in 2% (vol/vol) neutral buffered formalin, and incubated overnight at room temperature. Cells were washed extensively with PBS and resuspended at a concentration of 4 × 10^9^/mL. Anti‐UK1 antisera were raised in rats by Pocono Rabbit Farm and Laboratory.

### 2.6. Staining and Imaging of Monolayers

Membranes were cut from the transwell with a scalpel and permeabilized in 0.1% Triton X‐100 in PBS for 15 min. Membranes were washed three times with PBS and blocked by incubation in 2% BSA and 1% normal donkey serum in PBS for 1–2 h. Probing with primary antibodies in 2% BSA using manufacturer‐recommended dilutions was conducted at 4°C overnight. Primary antibodies include the following: anti‐NHE3 (Novus NBP1‐82574, 1:100), anti‐MUC2 (GeneTex GTX100664, 1:200), anti‐*β*‐catenin (Invitrogen 13‐8400, 1:100), anti‐CCL20 (Invitrogen PA5‐47517, 1:20), and antiformalin fixed UK1. Membranes were washed three times with PBS and probed with secondary antibodies (donkey antirat Alexa Fluor 488 [Invitrogen A21208, 1/1000], donkey antirabbit Alexa Fluor 488 [Invitrogen A21206, 1/1000], donkey antirabbit Alexa Fluor 594 [Invitrogen A21207, 1/1000], donkey antimouse Alexa Fluor 594 [Invitrogen A21203, 1/1000], or donkey antigoat Alexa Fluor 594 [Invitrogen A32758, 1/1000]) in 2% BSA for 1–2 h. Membranes were washed with PBS containing Hoechst 33342 (Invitrogen H3570, 1/1000) and, for some experiments, phalloidin‐Alexa Fluor 680 (Invitrogen A22286, 1/1000).

Membranes were imaged using a Leica DMi8 with a 63× oil immersion objective. Images were collected using LasX software. Image analysis and figure construction were performed using Fiji. Images shown of the *X* *Y* plane are maximum intensity *Z* projections. Orthogonal views were analyzed using LasX. Bacteria were defined as in a vertical orientation if the long axis of the bacterium was perpendicular to the long axis of the monolayer (90^°^ ± 45^°^) in both the *X* *Z* and *Y* *Z* planes.

### 2.7. Single‐Cell RNA Sequencing (scRNA‐Seq)

Monolayers were cultured on 12‐mm‐diameter, 0.4‐*μ*m polycarbonate filters (Millicell PIHP01250) and differentiated as described above. Spores were treated with glycine and sodium cholate, as above, collected by centrifugation (Eppendorf 5415, 13,200 rpm, 5 min), and resuspended in PBS, and 1 × 10^5^ spores in 10 *μ*L of PBS was added to the apical medium. Transwells were incubated as described above.

After 4, 15, 20, or 25 h of incubation, monolayers were washed with 100 *μ*L PBS, EDTA 0.5 mM, pH 8, and incubated with TrypLE Express (600 *μ*L in the basolateral and 400 *μ*L in the apical compartments) at 37°C for 12 min.

One hundred microliters 50% FBS was added to the apical compartment, and the cells were suspended by pipetting up and down several times. Cells were passed through a 40‐*μ*m cell strainer and recovered in tubes precoated with 0.1% BSA. The transwells were then washed with 400 *μ*L CMGF‐, which was passed through the strainer and combined with the previous filtrate. Cells were collected by centrifugation at 300 g × 5 min. Most of the supernatant was gently removed. Cells were resuspended in 1 mL of 0.04% BSA and 5 *μ*M Y27632 and collected as above. Using a wide‐bore tip, the pellet was resuspended in the remaining supernatant by pipetting up and down. Cells were counted with a Corning CytoSMART counter and adjusted to 1 × 10^6^ cells/mL.

The remaining steps were performed following the manufacturer′s protocol. Briefly, the cell suspension was loaded, along with reagents, barcoded Single Cell 3 ^′^ v3.1 Gel Beads, and Partitioning Oil, onto the Chromium Next GEM Chip G. Cells were partitioned into GEMS using the Chromium Controller. Gel beads were then dissolved, releasing primers, and the cells were lysed. Reverse transcription was performed to generate cDNA tagged with cell‐specific barcodes and Illumina TruSeq Read 1 sequencing primer. GEMS were broken, and the first‐strand cDNA was purified and PCR‐amplified. After enzymatic fragmentation followed by size selection, the cDNA was PCR‐amplified, incorporating a sample index and primers P5, P7, and TruSeq Read 2 for Illumina sequencing. Library quality was assessed using an Agilent Bioanalyzer. Paired‐end sequencing was performed by the Tufts University Genomics Core Facility.

### 2.8. scRNA‐Seq Data Analysis

Initial data analysis, including demultiplexing, barcode processing, and aligning reads to the human genome, was performed using Cell Ranger. Seurat Version 5.0.2 [33] was used to analyze the scRNA‐seq dataset. Transcriptomes with fewer than 250 or more than 5000 detected expressed genes or more than 40% mitochondrial expressed genes were removed from the analysis. The *C. difficile* toxins TcdA and TcdB are cytotoxic, and we, therefore, expected that some responding cells would be unhealthy. We analyzed cells with up to 40% mitochondrial expressed genes to retain some of the less healthy cells. Gene expression profiles of two replicates of mock and CDI colonoid monolayers were log‐normalized, scaled, and subjected to dimensionality reduction with PCA (30 PCs). Results were integrated using Harmony integration (30 PCs) to mitigate sample variability and group transcriptionally similar cells together. Clustering using the Louvain algorithm was conducted at resolution 0.1, yielding four clusters.

Cluster markers were identified using FindAllMarkers() with logfc.threshold = 0.25, min.pct = 0.1, and only.pos = TRUE to compare gene expression in cells of one cluster to all other cells in the other three clusters. The markers obtained were compared to previously described markers for colonic cell types [34] from the Gene Set Enrichment Analysis (GSEA) Human Molecular Signatures Database. SeuratExtend was used to produce dot plots [35].

Cell type annotation was conducted using SingleR [36], a reference‐based method for annotating cells that compares individual cell transcriptional profiles with the transcriptomes of reference cells. Colonoid cells were assigned the identity of the reference cell that was most comparable using the human colon reference published by Smillie et al. [37], which included 12 healthy colon biopsy samples. After annotation, cell type counts were exported for analysis in Excel. Related cell types were combined into groups to simplify the findings. The following labels were used: Enterocytes = Enterocytes + Immature Enterocytes 1 + Immature Enterocytes 2; Goblets = Goblets + Immature Goblets; M‐like cells = M cells; and Other = Stem + TA 1 + TA2 + Tuft + NA. Several cell types were grouped into the “Other” category because of their very low abundance. GraphPad Prism was used to produce graphs of cell type composition.

Differentially expressed genes upregulated or downregulated in *C. difficile* cocultures versus mock samples were identified for each cluster using the Seurat FindMarkers() function (logfc.threshold = 0.25 and min.pct = 0.10).

### 2.9. RNA Extraction and Real‐Time RT‐qPCR

Colonoid cells (4–5 × 10^5^ cells/transwell) were seeded on 12‐mm‐diameter, 0.4‐*μ*m polycarbonate filters and incubated for 5 days. Medium was changed to DM, and the transwells were incubated for another 5 days. Primocin was withdrawn 2 days before inoculation with pregerminated UK1 spores.

Pregerminated UK1 spores (1 × 10^6^) or buffer were added to each transwell. After incubation for 16 h, the transwell was moved to an empty well, the apical medium was removed, and the membrane was washed. TRIzol (0.3 mL) was added to the apical compartment, pipetted up and down five times, and transferred to a tube. A second 0.3 mL of TRIzol was added and allowed to incubate briefly. After pipetting up and down, the second TRIzol extract was combined with the first and frozen at −80°C. RNA was extracted using the Purelink RNA mini kit (Invitrogen 12183025) with on‐column DNase treatment (Invitrogen 12185‐010) following the manufacturer′s protocol. An aliquot of RNA was used to generate cDNA by reverse transcription with Superscript III (Invitrogen 18080044) following the manufacturer′s protocol. cDNA was diluted 1:3 and used as a template for RT‐PCR reactions.

*CCL20*, *ANKRD37*, and *GAPDH* gene expression was measured in cDNA by qPCR using 2× SYBR Green MasterMix (Applied Biosystems 4334973). qPCR reactions were run on a Roche LightCycler 480 II as described [38]. *CCL20* and *ANKRD37* primer sequences (from Harvard PrimerBank) were as follows: hCCL20 F1 5 ^′^ TGCTGTACCAAGAGTTTGCTC 3 ^′^, hCCL20 R1 5 ^′^ CGCACACAGACAACTTTTTCTTT 3 ^′^, hANKRD37 F1 5 ^′^ TTAGGAGAAGCTCCACTACACAA 3 ^′^, and hANKRD37 R1 5 ^′^CACTGGCTACAAGCAGGCT 3 ^′^. *GAPDH* primer sequences were designed using Primer‐BLAST: hGAPDH F1 5 ^′^ CATGTTCGTCATGGGTGTGAA 3 ^′^ and hGAPDH R1 5 ^′^ GACTGTGGTCATGAGTCCTTCC 3 ^′^. Primer specificity was demonstrated by Sanger sequencing of the qPCR product. No template controls yielded no signal. Gene expression relative to the average of mock‐inoculated samples was calculated by the 2^−*Δ* *Δ*CT^ method. [39].

## 3. Results

### 3.1. A Model of the Human Intestinal Epithelium Derived From Human Colonoids

**Figure 1:**
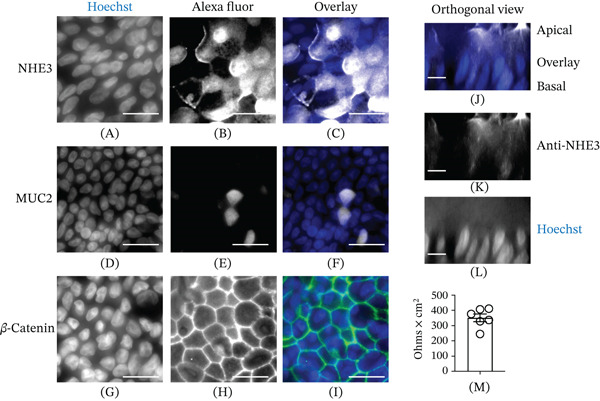
Colonoids differentiated on polycarbonate filters in transwells produce monolayers of polarized, differentiated cells with barrier function. Colonoids were cultured in Matrigel, harvested, dissociated, and seeded on polycarbonate filters in transwells. After growth to confluency and incubation in DM for 5 days, the monolayers were fixed with 4% PFA. Fixed monolayers were permeabilized and stained with antibody and Hoechst as described in the Materials and Methods. Immunofluorescent signal was detected using a Leica DMi8 microscope and visualized with a 63× oil immersion objective. (A, C [blue], D, F [blue], G, I [blue]) Staining with Hoechst; (B, C [white]) staining with anti‐NHE3; (E, F [white]) staining with anti‐MUC2; (H, I [green]) staining with anti‐*β*‐catenin. Scale bar, 20 *μ*m. (J–L) Orthogonal views of the *Z* plane showing polarization. (J, L [blue]) Staining with Hoechst; (K, L [white]) staining with anti‐NHE3. Scale bar, 10 *μ*m. (M) Transepithelial electrical resistance was measured as described in the Materials and Methods. Each symbol shows results from a different monolayer, bar shows the mean with the standard error of the mean.

Differentiated human colonoid monolayers were used as a model of the intestinal epithelium. To produce monolayers for coculture with bacteria, the colonoids were enzymatically disaggregated and seeded onto Matrigel‐coated polycarbonate transwell filters. The monolayers were grown to confluence and then incubated for 5 days in DM. Immunofluorescent staining of fixed, differentiated monolayers showed that markers of differentiated epithelial cells, such as NHE3 (sodium–proton exchanger, marker of colonocytes; Figure 1B,C) and MUC2 (mucin, marker of goblet cells; Figure 1E,F), were expressed. The cells were polarized with nuclei localized toward the basal side (Figure 1J–L). Figure 1C,F overlays show the apical side of the monolayer with the basal side below. Apical markers, therefore, obscure the nuclei in some regions of the images. Further, the cells formed intercellular junctions (detected by *β*‐catenin staining of adherens junctions; Figure 1H,I), and TEER values ranging from 300 to 450 *Ω* cm^2^ were typically obtained (Figure 1M). This model of the human intestinal epithelium thus recapitulated many features of the human colonic epithelium.

### 3.2. Human Colonoid Monolayers Support the Growth of the Anaerobic Pathogen *C. difficile*

We tested whether this model colonic epithelium would support the growth of the anaerobic bacterium *C. difficile* in coculture in aerobic conditions. Monolayers were incubated without antibiotics before inoculation with *C. difficile*. Spores of *C. difficile* strain UK1 [25] were pregerminated and added to the apical compartment of the transwell (5 × 10^5^ spores/transwell). Mock‐inoculated cultures received buffer. The cocultures were incubated in a standard tissue culture incubator with a 5% CO_2_, room air atmosphere.

**Figure 2:**
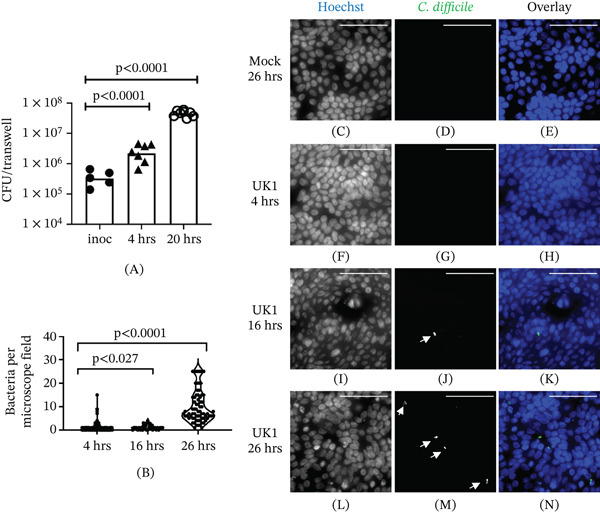
Differentiated colonoid monolayers support the growth of *C. difficile.* Differentiated colonoid monolayers were produced as shown in Figure 1. (A) Monolayers were inoculated with pregerminated spores of *C. difficile* strain UK1. Total CFU/transwell detected in the apical culture medium is shown. Each symbol shows results from a different transwell. Bar, geometric mean. *p* values, one‐way ANOVA performed with log‐transformed data, using Dunnett′s multiple comparison test. Composite results from three experiments. (B) Bacteria per microscope field were scored visually and counted. Each symbol shows results from one 63× microscope field. Four hours, *n* = 201 fields, median = 0; 16 h, *n* = 34 fields, median = 1; 26 h, *n* = 71 fields, median = 7; combined results from two to three experiments. *p*, Kruskal–Wallis with Dunn′s multiple comparisons test. (C–N) Monolayers were inoculated with pregerminated spores of *C. difficile* strain UK1 or buffer (mock). Monolayers were incubated for (F–H) 4 h, (I–K) 16 h, or (C–E, L–N) 26 h after inoculation. After removing the apical medium and washing the apical surface as described in the Materials and Methods, epithelial monolayers were fixed in 4% PFA and stained for immunofluorescence. (C, E [blue], F, H [blue], I, K [blue], L, N [blue]) Stained with Hoechst; (D, E [green], G, H [green], J, K [green], M, N [green]) stained with anti–*C. difficile*. White arrows indicate bacteria. Scale bar, 50 *μ*m.

After incubation, the number of viable *C. difficile* bacteria in the cocultures was estimated by sampling the apical medium of the transwell. The inoculated transwells were transferred to the anaerobic chamber, and the most apical medium, expected to be more aerobic, was removed and discarded; this sample was not turbid, consistent with low bacterial density. The lower apical medium, next to the monolayer, was carefully collected into a pipette tip and used to wash the monolayer gently two times; this sample was typically turbid, consistent with higher bacterial density. The apical culture medium was transferred into a prereduced, preweighed Eppendorf tube, and a sample was immediately diluted with prereduced PBS and further diluted for plating on prereduced BHIS plates. Colonies were enumerated, and the total CFU/transwell was calculated. Results showed an increase in CFU/transwell after 4 h of coculture (4 h; Figure 2A) or 20 h of coculture (20 h; Figure 2A) compared to the inoculum (inoc; Figure 2A). *Clostridioides difficile* bacteria thus grew and survived in coculture with colonoid monolayers despite the aerobic atmosphere of the incubator.

### 3.3. *Clostridioides difficile* Associates With the Epithelium and Exhibits Vertical Orientation

In addition to proliferating, bacteria became associated with the monolayer during coculture. After monolayers were washed as described above for enumeration of *C. difficile* CFU, the transwells were removed from the anaerobic chamber and fixed with 4% paraformaldehyde, followed by washing with PBS. The polycarbonate filter was cut from the transwell and incubated for immunofluorescence as described in the Materials and Methods. Filters were stained with anti–*C. difficile* antiserum, Hoechst, and anti‐NHE3. The number of bacteria associated with the colonoid monolayers was scored. After 4 h of coculture, the median number of bacteria per 63× field was 0; after 16 h, the median was 1; and after 26 h, the median was 7 (Figure 2B–N; bacteria marked with white arrows). No bacteria were detected in association with mock‐inoculated monolayers (Figure 2C–E). These results show that bacteria associated with the monolayer and their number increased over time.

**Figure 3:**
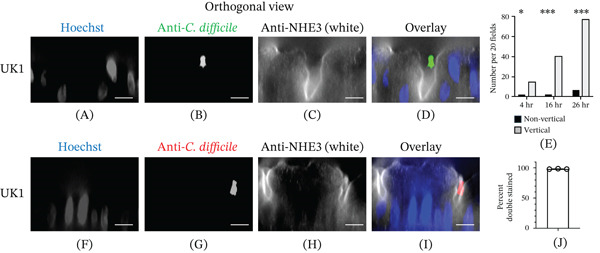
Monolayer‐associated *C. difficile* bacteria were extracellular and in a vertical orientation relative to the monolayer. Differentiated colonoid monolayers were produced as shown in Figure 2. (A–D) Monolayers were cocultured with pregerminated UK1 spores for 16 h, washed, fixed with 4% PFA, permeabilized, and stained. Orthogonal views are shown. The apical surface is above, while the basal surface is below. (A, D [blue]) Stained with Hoechst; (B, D [green]) stained with anti–*C. difficile*; (C, D [white]) stained with anti‐NHE3. Scale bar = 10 *μ*m. (E) Bacterial orientation was scored visually. A vertical bacterium was defined as a bacterium with the long axis at a 90° (±45°) angle relative to the long axis of the monolayer in both the *X* *Z* and *Y* *Z* planes. Seventeen to 19 63× microscope fields were scored, and numbers were normalized to 20 fields. Grey bars show vertical bacteria; black bars show nonvertical bacteria. Results tested against a 50:50 distribution by Fisher′s exact test.  ^∗^ *p* < 0.03 and  ^∗∗∗^ *p* < 0.0001. (F–I) Monolayers inoculated with pregerminated spores of strain UK1 were incubated for 20 h, washed, and fixed with 4% PFA. Before permeabilization, bacteria were stained with anti–*C. difficile* primary antibody and Alexa Fluor 594–conjugated secondary antibody (red). Monolayers were then permeabilized with 0.1% Triton X‐100 and stained with anti–*C. difficile* primary antibody and Alexa Fluor 488–conjugated secondary antibody (green). (F, I [blue]) Stained with Hoechst; (G, I [red]) stained with anti–*C. difficile* prior to permeabilization; (H, I [white]) stained with anti‐NHE3. Scale bar = 10 *μ*m. (J) Bacterial staining was scored visually (60–150 bacteria scored per experiment; three experiments), and the percentage of bacteria that were double‐stained was plotted. Over 98% of bacteria were double‐labeled. Rare bacteria that stained only after permeabilization (green only) were detected. No bacteria were labeled with red only.

Orthogonal views of the colonoid images demonstrated that the majority of monolayer‐associated *C. difficile* exhibited a vertical orientation. Images of the washed monolayers used in Figure 2 were analyzed in the *X* *Z* and *Y* *Z* planes, and bacterial orientation was scored visually. A bacterium was defined as vertical if its long axis was at a 90° (±45°) angle to the long axis of the monolayer in both *X* *Z* and *Y* *Z* planes. Figure 3A–D shows a vertically oriented bacterium in relation to the NHE3‐stained apical membrane of the monolayer. Quantification of vertical and nonvertical bacteria after 4, 16, or 26 h of coculture showed significant enrichment for vertical bacteria at all time points (Figure 3E; Fisher′s exact test vs. 50:50 distribution of bacteria).

Bacterial accessibility to antibody in the absence of epithelial permeabilization was analyzed to test whether monolayer‐associated bacteria were extracellular. Monolayers were cocultured with pregerminated UK1 spores for 20 h. Washed, fixed monolayers were stained with anti–*C. difficile* without permeabilization, followed by incubation with Alexa Fluor 594–conjugated secondary antibody (red; Figure 3G,I). The monolayers were then permeabilized and stained with anti–*C. difficile*, followed by incubation with Alexa Fluor 488–conjugated antibody (green). Most of the bacteria were double‐labeled (98%; Figure 3J), indicating accessibility to the antibody without permeabilization. Rare bacteria were stained only green. In these cases, a nearby bacterium appeared to be interfering with staining. No bacteria were stainedred only. These results show that monolayer‐associated bacteria were typically found vertically oriented in an extracellular location.

### 3.4. Identification of CCL20 as a Marker of Early Colonoid Responses to *C. difficile* Challenge

We conducted a scRNA‐seq study of colonoids in coculture with *C. difficile* or with buffer only (referred to as mock). A more detailed analysis of the responses of colonoids to *C. difficile* challenge over a time course will be presented elsewhere. Our analysis here focused on identifying gene markers that could be used to monitor the response of colonoids to *C. difficile* in coculture.

Differentiated colonoid monolayers were produced as described for Figure 1 and inoculated with pregerminated spores of strain UK1 (or buffer). Two replicates of the experiment comparing a Cd coculture with a mock culture after 15 h of incubation were performed, yielding four samples: Mock Replicate a (Ma), Mock Replicate b (Mb), *C. difficile* Coculture Replicate a (CDa), and *C. difficile* Coculture Replicate b (CDb).

As described in the Materials and Methods, single‐cell suspensions were produced and processed for scRNA‐seq using the 10× platform. Sequencing was conducted by the Tufts University Genomics Core Facility. Sequences were demultiplexed and aligned to the human genome using Cell Ranger. Results were analyzed using Seurat 5.0.2 [33] in R. Transcriptomes with fewer than 250 or more than 5000 detected expressed genes or more than 40% mitochondrial expressed genes were removed from the analysis. Transcriptomes with up to 40% mitochondrial expressed genes were included because *C. difficile* toxins are cytotoxic and some responding cells were expected to show signs of damage.

**Figure 4:**
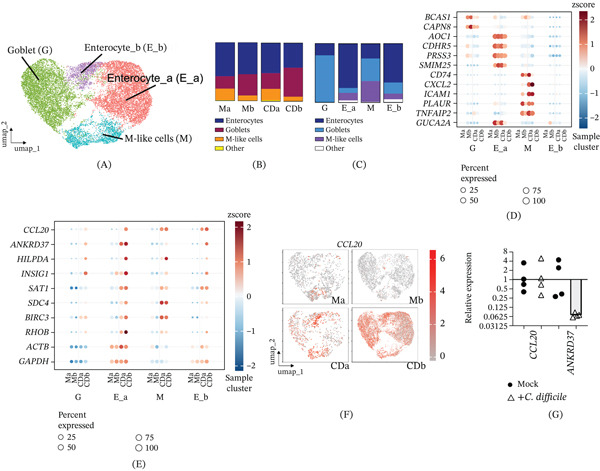
Markers expressed by colonoids during coculture with *C. difficile.* Differentiated colonoid monolayers were produced as shown in Figure 2. Monolayers cocultured with *C. difficile* or PBS for 15 h were harvested and subjected to single‐cell RNA‐sequencing (scRNA‐seq). Results were analyzed using Seurat as described in the Materials and Methods. (A) Cells are shown in a UMAP organized based on transcriptional similarity. Clusters are indicated with colors and labeled based on the dominant cell type in the cluster, as shown in panel C. (B) Cell type composition of each sample is shown. Ma, Mock Replicate a; Mb, Mock Replicate b; CDa, *C. difficile* Coculture Replicate a; CDb, *C. difficile* Coculture Replicate b. (C) Cell type composition of each cluster is shown. Others include stem cells, TA cells, and tuft cells. (D) Expression of cluster markers is shown in each cluster as a dot plot where the size of the dot indicates the percentage of the cluster expressing the gene and the color indicates the *z*‐score. *BCAS1* and *CAPN8*, markers of goblet cells. *AOC1*, *CDHR5*, *PRSS3*, *SMIM24*, and *GUCA2A*, markers of colonocytes. *CD74*, *CXCL2*, *ICAM1*, *PLAUR*, and *TNFAIP2*, markers of Paneth‐like cells/M cells. (E) Expression of genes is shown in each cluster as a dot plot, where the size of the dot indicates the percentage of the cluster expressing the gene and the color indicates the *z* score. *CCL20*, *ANKRD37*, *HILPDA*, *INSIG1*, *SAT1*, *SDC4*, *BIRC3*, and *RHOB* are candidates for markers of colonoid response to *C. difficile* coculture. *ACTB* and *GAPDH*, widely used housekeeping genes, are shown for comparison. (F) Feature plot indicating expression of *CCL20* in each cell in each sample. Grey indicates low expression, while shades of red indicate higher expression. (G) Additional differentiated colonoid monolayers were produced as shown in Figure 2 and cocultured with *C. difficile* (open triangles) or PBS (black circles) for 16 h. Medium was removed, and monolayers were extracted with TRIzol and the PureLink RNA purification kit as described in the Materials and Methods. *CCL20* or *ANKRD37* gene expression, normalized with *GAPDH* gene expression, was measured in cDNA by real‐time RT‐qPCR. Results are shown relative to the geometric mean of mock samples. Each symbol shows results obtained with one monolayer. Bar, geometric mean.

Transcriptomes were processed as described in the Materials and Methods, integrated using Harmony integration, and displayed in a UMAP based on their transcriptional similarity. Clustering using the Louvain algorithm yielded four clusters (Figure 4A). Transcriptomes with high mitochondrial content (up to 40%) were found in multiple clusters (Figure S1).

To identify cell types, a reference‐based method was employed using the SingleR package in R [36] with a reference based on an atlas of cells from healthy human colons [37]. The majority of the cells were annotated as enterocytes or goblet cells of varying maturity (Figure 4B,C). These results are consistent with immunofluorescence results (Figure 1) showing that cells positive for NHE3, a marker of colonocytes, or MUC2, a marker of goblet cells, were readily detected. Some cells were annotated as rarer cell types, including M cells, transit‐amplifying cells, stem cells, and tuft cells. Each sample contained the major cell types detected (Figure 4B). Clusters were given names based on their most abundant predicted cell type (Figure 4A,C). Cluster M was composed of a mixture of cells that were labeled enterocytes, goblets, and M‐like cells and was named based on its most distinctive cell type.

Analysis of genetic markers for each cluster provided further support for the cell type labels. Genes expressed more highly in each cluster (cluster markers) relative to cells in all other clusters were identified. Some of the cluster markers were also previously described as markers of colonic cell types [34]; expression of a subset of these genes is shown in Figure 4D (*BCAS1* and *CAPN8*, markers of goblet cells; *AOC1*, *CDHR5*, *PRSS3*, *SMIM24*, and *GUCA2A*, markers of colonocytes; and *CD74*, *CXCL2*, *ICAM1*, *PLAUR*, and *TNFAIP2*, markers of cells labeled “Paneth‐like cells” by Gao et al. [34] or M cells by Smillie et al. [37]). For most markers, cluster‐specific upregulation in all samples was observed. Cells of cluster E_b did not show strong expression of cluster‐specific markers despite being mostly labeled as “Enterocytes.”

To identify markers of colonoid responses to *C. difficile* in coculture, differential gene expression analysis comparing mock and Cd samples in each cluster was conducted using FindMarkers() with a log‐fold‐change threshold set to 0.25 and a minimum fraction of cells expressing a gene at 0.1. Results were ranked by adjusted *p* value, and the top candidate gene for every cluster was *CCL20* (Table S1). Markers that were expressed more highly in Cd samples and were among the Top 50 candidate genes (based on adjusted *p* value) in at least three of the four clusters were identified. Expression of these candidate genes is shown in Figure 4E. *RHOB* was included because it was upregulated in two of the four clusters and had been previously identified as differentially expressed in response to *C. difficile* [15, 17, 40, 41]. Widely used housekeeping genes *ACTB* (encoding actin) and *GAPDH* were included for comparison (Figure 4E). *CCL20* showed strong upregulation in response to *C. difficile* in both samples, and upregulation was detected in cells of all clusters (Figure 4D,E). *ANKRD37*, *HILPDA*, *INSIG1*, *SAT1*, *SDC4*, *BIRC3*, and *RHOB* showed upregulation in multiple clusters. Additionally, there were some genes that exhibited upregulation in one or a few clusters only. For example, *CXCL8*, encoding IL‐8, was upregulated most consistently in cluster M (Table S1). Thus, several candidates for *C. difficile*–responsive genes were identified, and *CCL20* was the top candidate. *CCL20* expression has been previously detected in response to *C. difficile* bacteria [24], supporting the results of this analysis.

Expression of *CCL20* and *ANKRD37* was further tested in colonoid monolayers by real‐time RT‐qPCR to determine whether upregulation could be detected using bulk RNA. Monolayers were produced and cocultured with or without *C. difficile* for 16 h as above. RNA was extracted, and real‐time RT‐qPCR was conducted as described in the Materials and Methods. As shown in Figure 4G, increased expression of *CCL20* or *ANKRD37* was not detected. The discrepancy between the results of the scRNA‐seq experiment and the analysis of bulk RNA may reflect heterogeneity in the responses of individual cells and the fact that the transcriptomes used for scRNA‐seq were heavily filtered (> 250 and < 5000 detected expressed genes and < 40% mitochondrial expressed genes), so only the healthiest cells were included in the analysis. In contrast, bulk RNA extraction included all cells in the monolayer, regardless of their health.

### 3.5. CCL20 Production During *C. difficile* Coculture Detected by Immunofluorescence

**Figure 5:**
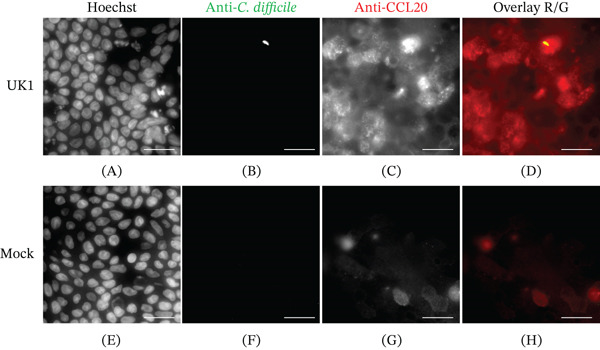
Increased production of CCL20 in monolayers cocultured with *C. difficile.* Differentiated colonoid monolayers were produced as shown in Figure 2. Monolayers were inoculated with pregerminated spores of (A–D) *C. difficile* strain UK1 or (E–H) buffer and cocultured for 16 h. Brefeldin A (25 *μ*g/mL final concentration) was added to the apical and basal media to block protein secretion, and monolayers were incubated for an additional 4 h. Medium was removed, and monolayers were washed, fixed in 4% PFA, and stained for immunofluorescence. (A, E) Staining with Hoechst; (B, D [yellow], F, H) staining with anti–*C. difficile*; (C, D [red], G, H [red]) staining with anti‐CCL20. Scale bar, 20 *μ*m. CCL20 production was observed in numerous cells throughout the cocultured monolayer.

To confirm the results of the scRNA‐seq and determine whether CCL20 was useful as a marker of colonoid responses to *C. difficile* in coculture, we employed immunofluorescence. Mock and *C. difficile* cocultured colonoids were incubated for 16 h. CCL20 is a secreted protein, and therefore, monolayers were treated with Brefeldin A (25 *μ*g/mL final concentration) for 4 h to block protein secretion and enhance the ability to detect cells producing CCL20. Monolayers were washed, fixed with 4% PFA, and probed with anti‐CCL20 antibody, anti–*C. difficile* antibody, and Hoechst. Figure 5A–D shows that bright CCL20 staining was observed in *C. difficile* cocultures in contrast to mock‐inoculated cultures (Figure 5E–H). The overlays shown in Figure 5D,H include only the red channel (CCL20) and the green channel (*C. difficile*). These results thus demonstrated increased CCL20 production in *C. difficile* cocultured colonoids, confirming the scRNA‐seq findings.

In some cases, CCL20‐positive cells were located near a detected *C. difficile* bacterium (Figure 5D). However, many responding cells lacked bacteria in their immediate vicinity at the time of imaging. Bacteria loosely associated with the monolayer may have been present during coculture but were washed away during processing. Additionally, colonoids may be responding to secreted bacterial products such as toxins, and thus, nearby bacteria would not be required for CCL20 upregulation. The role of toxins in *C. difficile*–dependent production of CCL20 was, therefore, tested further.

### 3.6. Nontoxigenic *C. difficile* Have Reduced Ability to Promote CCL20 Production

As discussed above, the secreted glucosylating toxins, TcdA and TcdB, are major virulence factors of *C. difficile*. Therefore, to identify the contribution of these toxins to bacterial/colonoid interactions, we examined the behavior of nontoxigenic *C. difficile* in the colonoid coculture system. TcdA and TcdB are encoded in the pathogenicity locus along with the sigma factor TcdR, which is required for toxin expression [9]. The nontoxigenic R20291::*tcdR* mutant [27] was compared to its toxigenic parent strain R20291 (an epidemic strain closely related to UK1 [26]).

**Figure 6:**
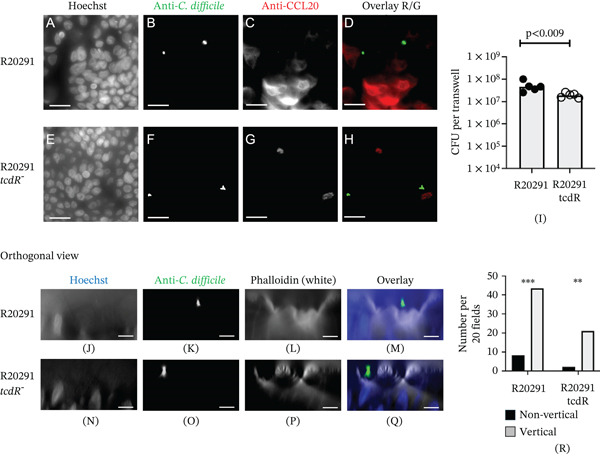
Reduced production of CCL20 by monolayers cocultured with nontoxigenic *C. difficile*. Differentiated colonoid monolayers were produced as shown in Figure 2. Monolayers were inoculated with vegetative cells of strain (A–D) R20291 or (E–H) R20291 *tcdR* and cocultured for 16 h. Brefeldin A (25 *μ*g/mL final) was added to the apical and basal media, and monolayers were incubated for 4 h. Medium was removed, and monolayers were washed, fixed in 4% PFA, and stained for immunofluorescence. (A, E) Staining with Hoechst; (B, D [green], F, H) staining with anti–*C. difficile*; (C, D [red], G, H [red]) staining with anti‐CCL20. Scale bar, 20 *μ*m. (I) Apical culture medium was collected in an anaerobic chamber, and total CFU/transwell was determined. Each symbol shows results from a different transwell. Bar, geometric mean. *p* values, *t* *-*test performed with log‐transformed data. Composite results from two experiments. Monolayers were inoculated with vegetative cells of strain (J–M) R20291 or (N–O) R20291::*tcdR* and cocultured for 17 h. Brefeldin A was not used. Monolayers were washed, fixed with 4% PFA, permeabilized, and stained. Orthogonal views are shown with the apical surface above and the basal surface below. (J, M [blue], N, Q [blue]) Stained with Hoechst; (K, M [green], O, Q [green]) stained with anti–*C. difficile*; (L, M [white], P, Q [white]) stained with phalloidin to stain filamentous actin. Scale bar = 10 *μ*m. (R) Bacterial orientation was scored as shown in Figure 4, and numbers were normalized to 20 microscope fields. Grey bars show vertical bacteria; black bars show nonvertical bacteria. Results tested against a 50:50 distribution by Fisher′s exact test.  ^∗∗^ *p* < 0.004 and  ^∗∗∗^ *p* < 0.0004.

Transwells containing differentiated colonoid monolayers were produced as described above and inoculated with either R20291, R20291::*tcdR*, or buffer. These transwells were inoculated with vegetative cells because spores of strain R20291::*tcdR* show an altered response to germinants [27]. After 17 h of coculture, the apical medium was collected, used to wash the monolayer, diluted, and plated to enumerate *C. difficile* CFU as described in the Materials and Methods. Vegetative cells of both strains grew in the colonoid coculture system, reaching levels of 2–4 × 10^7^ CFU/transwell (Figure 6I). Glucosylating toxin production was thus not required for bacterial growth in this system, although the *tcdR* mutant strain reached a level approximately twofold lower than the WT strain. The *tcdR* mutant did not exhibit a defect in growth or saturation density under anaerobic conditions [27].

To test for CCL20 upregulation in colonoids, monolayers were cocultured with R20291 or R20291::*tcdR* for 16 h. Brefeldin A was added to the apical and basal medium, and the monolayers were incubated for four more hours. Monolayers were washed, fixed, permeabilized, and incubated with antibody or stains for fluorescence microscopy. Colonoids cocultured with WT R20291 showed expression of CCL20 (Figure 6A–D), while colonoids cocultured with R20291::*tcdR* did not show this response (Figure 6E–H). The overlays shown in Figure 6D,H include only the red channel (CCL20) and the green channel (*C. difficile*). Thus, nontoxigenic bacteria did not promote production of CCL20 to the same extent as WT bacteria. These observations suggest that secreted toxins play a role in activating widespread responses to *C. difficile* in the colonoid monolayer system. Consistent with these results, previous studies demonstrated CCL20 upregulation by purified TcdB or *C. difficile* culture supernatants [24, 42].

Despite the failure to promote CCL20 production, nontoxigenic *C. difficile* strains associated with the monolayer in a vertical orientation. Monolayers were cocultured with bacteria, with or without Brefeldin A, and then washed, fixed, permeabilized, stained, and imaged. Phalloidin staining of filamentous actin was used to identify the apical surface of the cells. As shown in Figure 6 J–R, most of the monolayer‐associated bacteria were in the vertical orientation for both strains. Bacteria also showed a vertical orientation after Brefeldin A treatment. Thus, toxin production and early colonoid responses to *C. difficile* challenge were not required for bacteria to associate with the monolayer in the vertical orientation.

## 4. Discussion

This study demonstrates that early interactions between *C. difficile* and the intestinal epithelium can be modeled in a simple tissue culture system using human colonoids. We found that *C. difficile* was able to grow in coculture with colonoid‐derived epithelial monolayers in standard tissue culture conditions (5% CO_2_, with ambient oxygen). In this simple system, bacterial association with the monolayer was detected after prolonged coculture. Further, colonoid responses to *C. difficile*, such as upregulation of CCL20, were observed at a time point when only small numbers of tightly associated bacteria were detected with the monolayer. Early upregulation of CCL20 may represent a response to production of glucosylating toxins by *C. difficile*, whereas bacterial association with the colonoid monolayer occurred independently of toxin production.

The observation that bacterial cells associated with the colonoid monolayer is consistent with previous studies demonstrating that *C. difficile* expresses proteins with adhesion functions [43]. For example, cell wall proteins Cwp2 [44] and Cwp66 [45] promote adherence to epithelial cells in vitro, and CD2831 [46] and CbpA [47] bind components of the mammalian extracellular matrix, such as collagen. These adhesion‐associated proteins may contribute to the binding of *C. difficile* bacteria to the colonoid monolayer during coculture. The vertical orientation of bacteria detected in this study may indicate that this orientation promotes a tighter association of bacteria with cells.

CCL20 was detected as a protein produced by colonoids in response to *C. difficile*. Previous studies have described similar upregulation of CCL20 in response to *C. difficile*. Live *C. difficile* bacterial culture or bacterial culture supernatant [24] or purified *C. difficile* toxin TcdB and flagella [42] upregulated CCL20; the effects of toxin and flagella were synergistic. In addition to CCL20, several other genes, including *ANKRD37*, *HILPDA*, and *INSIG1*, were identified as *C. difficile* toxin–responsive, differentially expressed genes in previous transcriptomic studies [15–17, 40, 41, 48]. The results reported here are consistent with these earlier observations and further demonstrate that toxins produced by live bacteria are sufficient to stimulate this response.

Additionally, a variety of other conditions have been shown to upregulate *CCL20* in host cells. Several pathogens, including *Salmonella typhimurium*, *Mycobacterium avium paratuberculosis*, *Salmonella enteritidis*, and *Listeria monocytogenes* [24, 49], and bacterial components, such as flagellin [49] and Hepatitis C virus [50], increase CCL20 production.

CCL20 upregulation has also been detected in inflammatory bowel disease (IBD) [51, 52]. IBD is a chronic condition characterized by abnormal inflammatory responses to normal microbiota. The responses evoked by excessive CCL20 production play an important role in this disease.

Only a small number of tightly associated bacteria were observed after 15 h of coculture. However, widespread responses to *C. difficile* were identified based on gene expression (Figure 4D,E) and detection of CCL20 (Figure 5). Notably, cells lacking any visibly adjacent bacteria also upregulated CCL20. This response was not triggered by nontoxigenic *C. difficile* (Figure 6E–H). Together, these results argue that secreted toxins act both locally, near toxin‐producing bacteria, and also at a distance, enabling widespread response throughout the monolayer. Given that bacterial populations exhibit heterogeneous expression of *tcdA* during infection [53], such widespread effects may help sustain the survival of bacterial cells that are not actively expressing toxin.

Previous studies have investigated *C. difficile*/epithelial cell interactions in tissue culture systems. One approach employs specialized apparatus to create an anerobic environment in the apical compartment and aerobic conditions in the basolateral compartment, thereby supporting simultaneous growth of bacteria and epithelial cells [10–13, 54].

Simpler systems have cocultured intestinal epithelial cells with *C. difficile* using short‐term anaerobic conditions [20] that are nonoptimal for the epithelial cells or high MOI under aerobic conditions [55] that are nonoptimal for the bacteria. To bypass the need for live bacteria entirely, other studies have examined the effects of individual *C. difficile* components such as TcdA [17], flagella [18], or membrane vesicles [19].

In the present study, we exploited the ability of colonoids growing in a transwell monolayer to support the growth of *C. difficile* in the apical medium above the monolayer. This coculture system utilizes standard transwell inserts, which are widely available and do not require specialized equipment to use. Advances in organoid culture have enabled major breakthroughs in understanding the pathogenicity of viruses and parasites that were previously uncultivable in tissue culture systems [56, 57]. Likewise, for *C. difficile*, the colonoid‐based coculture system offers considerable advantages for dissecting *C. difficile* virulence mechanisms, probing human epithelial responses, testing therapeutic interventions, and performing high‐resolution imaging of bacterial–epithelial interactions. Together, these benefits will help accelerate research into *C. difficile*–human epithelium interactions.

## 5. Conclusions

This communication describes a coculture system in which the strict anaerobe *C. difficile* proliferated with differentiated human colonoid monolayers derived from colonic biopsies and used to model the intestinal epithelium. Analysis of epithelial responses demonstrated upregulation of the chemokine CCL20 as a prominent early response to infection. These findings establish a human epithelial infection model and identify CCL20 activation as an early host response to *C. difficile* in this model, providing a framework for investigating mechanisms of CDI pathogenesis.

## Author Contributions

P.Z. and A.D.G. contributed equally to this work. Conceptualization, P.Z. and C.A.K.; methodology, P.Z.; formal analysis, A.D.G, A.T., R.B., and C.A.K.; investigation, P.Z., A.W.D., J.D., and C.A.K.; resources, R.G., M.A., and J.R.; writing—original draft preparation, P.Z., A.D.G., and C.A.K.; writing—review and editing, R.G., A.W.D., J.D., M.A., J.R., A.T., and R.B.; supervision, C.A.K.; project administration, C.A.K.; funding acquisition, C.A.K.

## Funding

This study was funded by the National Institutes of Health, 10.13039/100000002, U19AI131126, R21AI168849, and T32AI007422; and the Tufts University Data Intensive Studies Center.

## Conflicts of Interest

The authors declare no conflicts of interest.

## Supporting Information

Additional supporting information can be found online in the Supporting Information section.

## Supporting information


**Supporting Information 1** Figure S1: Mitochondrial gene expression in samples.

**Supporting Information 2** Table S1: *Clostridioides difficile–*responsive genes.

## Data Availability

scRNA‐seq data are deposited in the NCBI GEO database (accession number GSE327570).
